# Ruscogenin Prevents Folic Acid-Induced Acute Kidney Damage by Inhibiting Rev-erb*α*/*β-*Mediated Ferroptosis

**DOI:** 10.1155/2022/8066126

**Published:** 2022-07-08

**Authors:** Mingyue Hu, Songbo An

**Affiliations:** Department of Pharmacy, The First People's Hospital of Lianyungang, Lianyungang 222002, Jiangsu, China

## Abstract

To investigate the pharmacodynamic effects of ruscogenin on acute kidney injury and the Rev-erb*α*/*β* regulation of ferroptosis intervention mechanism. The C57BL-6 mice were induced acute kidney injury with folic acid. Plasma, urine, and kidney samples were collected after intraperitoneal injection of ruscogenin (0.01, 0.1, and 1 mg/kg). We measured mouse kidney function indicators, including creatinine (CRE), blood urea nitrogen (BUN), N-acetyl-*β*-D-glucosidase (NAG), albumin, albumin and creatinine rate (ACR), renal index, and renal injury molecule-1 expression. Meanwhile, we detected the levels of ferroptosis indicators malondialdehyde (MDA), carbonylated proteins, iron ions, glutathione peroxidase 4 (GPX-4), and glutathione (GSH). The expression of solute carrier family 7 member 11 (Slc7a11), heme oxygenase-1 (HO-1), and Rev-erb*α*/*β* were detected by the Western blot and quantitative reverse transcription polymerase chain reaction (qRT-PCR), respectively. Ruscogenin (1 mg/kg) significantly reduced the index of folic acid-induced acute kidney injury and alleviated acute kidney injury. In kidney tissues, ruscogenin inhibited folic acid-induced Rev-erb*α*/*β* expression, restored HO-1 and SLC7A11 expression to normal levels, and alleviated ferroptosis. Ruscogenin ameliorates acute kidney injury via suppressing ferroptosis in kidney tissues through modulation of the Rev-erb*α*/*β*-SLC7A11/HO-1 pathway.

## 1. Introduction

Acute kidney injury (AKI) is a condition in which there is a sudden decline in kidney function due to tubular ischemia, nephrotoxins, nephritis, or urinary tract blockage [1]. In hospitalized and seriously injured patients, AKI has an extraordinarily high morbidity and fatality rate [2]. Very few specific therapies other than dialysis are currently available for the treatment of AKI [3]. As a result, new ways for preventing and treating AKI are desperately needed.

AKI pathogenesis is exceedingly complex. A new study indicates that the cellular ferritic pathway has a role in the pathogenesis of AKI [4]. Ferroptosis is an iron-dependent form of cell death that is distinct from ordinary apoptosis. Increased lipid peroxidation characterizes ferroptosis, which is regulated by intracellular iron ion levels as well as proteins including xc-, GPX-4, and HO-1 [5, 6]. In a mouse model of folic acid-induced AKI, Rev-erb*α*/*β* has been shown to block nuclear transcription of Slc7a11 and HO-1, reduce the inhibitory force of iron death, and direct the development of kidney injury [7]. Rev-erba and Rev-erbb belong to nuclear receptor subfamily 1 D members that induce transcriptional repression of target genes and contribute in pathological processes such as inflammatory responses by binding to gene specific response elements in promoters [8]. Therefore, the development of AKI therapeutics using Rev-erb*α*/*β* as an intervention target is a highly promising strategy.

Ruscogenin is a natural drug extracted from the traditional Chinese herbal medicine maitake with anti-inflammatory and anti-injury activities [9]. It has shown great potential for application in the treatment of inflammatory injury diseases such as acute lung injury [10], acute proctitis [11], acute pancreatitis [12], and ischemia-reperfusion injury [13]. However, the mechanism of lusiganine's efficacy against acute kidney injury and iron toxicity has not been elucidated. So, the current study looked at quercetin's ability to trigger ferroptosis in breast cancer cells and investigate its mode of action to promote ferroptosis via the TFEB-lysosome pathway. It lays the groundwork for a better understanding of quercetin's anticancer mechanism.

## 2. Materials and Methods

### 2.1. Reagents

Sigma-Aldrich supplied the folic acid. Ruscogenin was purchased from MedChem Express. CRE, BUN, MDA, GSH, albumin, and carbonylated protein kits were got from the Nanjing Jiancheng Institute of Biological Engineering. NAG activity kits were purchased from Beijing Solaibao Technology Co. Anti-Slc7a11 (Santa Cruz, Santa Cruz, CA, USA), anti-HO-1 (Santa Cruz, Santa Cruz, CA, USA), anti-Rev-erb*α* (Sigma-Aldrich, St. Louis, MO, USA), anti-Rev-erb*β* (Abnova, Taipei, Taiwan, China), anti-GPX-4 (Santa Cruz, Santa Cruz, CA, USA), and anti-GAPDH (Cell Signaling Technology, Danvers, MA, USA) were used in the study. C57BL-6 mice were purchased from Nanjing Qinglong Mountain Animal Breeding Farm.

### 2.2. Mice

Thirty eight-week-old C57BL/6 mice were prepared and randomly grouped as six groups: blank, blank + ruscogenin (1 mg/kg), folic acid group, folic acid + low-dose ruscogenin (0.01 mg/kg) group, folic acid + medium-dose ruscogenin (0.1 mg/kg) group, and folic acid + high-dose ruscogenin (1 mg/kg) group. This study was approved by the Animal Ethics Committee of The First People's Hospital of Lianyungang Animal Center.

### 2.3. Preparation of the Acute Kidney Injury Model

C57BL/6 mice were given an intraperitoneal injection of folic acid solution, which resulted in an acute kidney injury model. The daily injectable dose was 100 mg/kg up to seven days. Food and water were provided during the induction of the acute kidney injury model. 24 hours after the last dose, blood is collected from the orbits of mice and stored in anticoagulation tubes. Mice are sacrificed to isolate the kidney tissue. The whole blood was centrifuged at 4000 rpm for 10 min with 4°C. The supernatant was stored at −20°C. Fresh kidney tissues were immediately lysed, and the tissue lysate was collected and stored at −20°C. Urine samples were collected for 24 hours before the end of the experiment using a metabolic cage.

### 2.4. The Measurement of Acute Kidney Injury

To begin, according to the commercial kit instructions, the kits should be withdrawn from the refrigerator 1 hour before the experiment to allow all of the reagents to return to room temperature for more stable results. CRE, MDA, BUN, NAG, albumin, carbonylated protein, iron, and GSH levels were tested using analytical kits as directed. Urine NAG activity, urinary ACR, and renal index (kidney weight/body weight) were all calculated.

### 2.5. Determination of mRNA Expression by qRT-PCR

Total RNA was extracted with the TRIzol reagent (TaKaRa, Shiga, Japan) and reverse transcribed into cDNA with the RT Master Mix (Vazyme, Nanjing, China). SYBR Green Master Mix was used for qRT-PCR (Vazyme, Nanjing, China).  Table 1 shows the primer sequences. As an internal control, procyclin b was used. The 2^−ΔΔCT^ approach was used to calculate relative mRNA levels.

### 2.6. Western Blotting Assay for Protein Expression

Tissue samples were lysed in 1M PMSF-containing cell lysate. The bicinchoninic acid (BCA) (Pierce, Rockford, IL, USA) was used to determine the protein content. For electrophoresis, equal amounts of protein samples (40 mg) were loaded into 10% sodium dodecyl sulphate-polyacrylamide gel electrophoresis (SDS-PAGE) gels and transferred to polyvinylidene fluoride (PVDF) membranes (Millipore, Billerica, MA, USA). After blocking with 5% nonfat milk in tris buffered saline-tween (TBST) for 1 h. The membranes were incubated with primary antibodies overnight at 4°C, followed by incubation with horse radish peroxidase (HRP)-conjugated secondary antibodies at room temperature for 1 h. The target protein was detected using an electrochemiluminescence (ECL) assay kit. All data were quantitatively analyzed by image J.

### 2.7. Statistical Analysis

The data were presented as the mean ± standard deviation (*n*= 5) and the Shapiro–Wilk test was used to determine normality. The Student's *t*-test was used to compare statistical differences between the two groups. Multiple group comparisons were performed using one-way or two-way ANOVA test (^*∗*^*P* < 0.05). All statistical analyses were performed using GraphPad Prism 7.0 (La Jolla, CA, USA) (^*∗*^*P* < 0.05, ^*∗*^*P* < 0.01, and ^*∗*^*P* < 0.001).

## 3. Results

### 3.1. Ruscogenin Inhibits Folic Acid-Induced AKI in Mice

**Figure 1:**
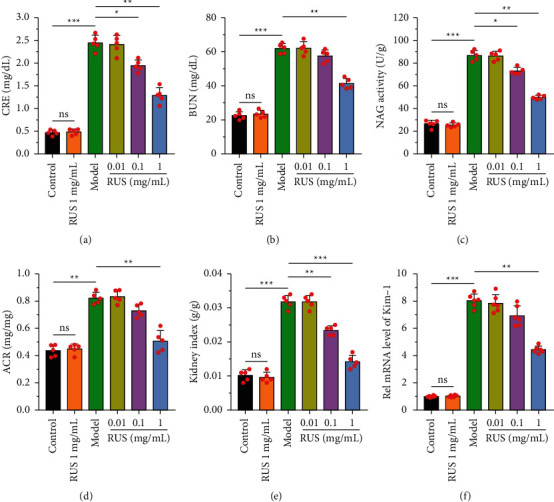
Changes in various kidney function indices in mice after treatment with ruscogenin. (a) Serum creatinine, (b) blood urea nitrogen, (c) *N*-acetyl-*β*-D-glucosidase activity, (d) urinary albumin creatinine ratio, (e) kidney index, and (f) Kim-1 expression was tested in each group. ^*∗*^*P* < 0.05,^*∗∗*^*P* < 0.01,^*∗∗∗*^*P* < 0.001.

The occurrence of AKI has been clinically well established for detection. Six typical measures of renal function were investigated and used to demonstrate the incidence of folic acid-induced AKI damage in mice. Serum creatinine (CRE), blood urea nitrogen (BUN), *N*-acetyl-*β*-D-glucosidase activity (NAG activity), urinary microalbumin-to-creatinine ratio (ACR), kidney index (kidney index), and Kim-1 were examined, respectively. According to the results of the study, there was no significant difference in the renal function index between the blank control and blank ruscogenin (1 mg/kg) treatment groups. After using folic acid to induce AKI for 7 days, the values of all test indexes were significantly elevated in mice, suggesting an extremely rapid decrease in kidney function. In each ruscogenin treated group, the kidney function indexes improved with the increase of ruscogenin concentration. Furthermore, there was no statistically significant difference between the 0.01 mg/kg ruscogenin group and the control group. In contrast, the best recovery from AKI was observed in the ruscogenin-treated group (1 mg/kg), with significant differences in all indices (Figure 1). The study indicated that ruscogenin (1 mg/kg) treatment significantly improved folic acid-induced AKI.

### 3.2. Ruscogenin Inhibits Folic Acid-Induced Ferroptosis in Kidney Tissues

**Figure 2:**
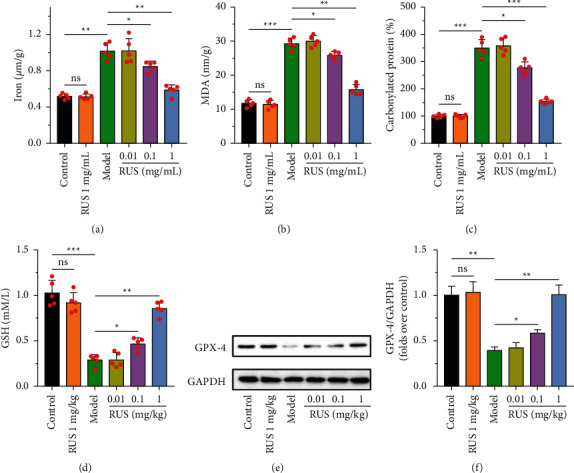
After treatment with ruscogenin, the iron ion content (a), malondialdehyde content (b), carbonylated protein content (c), and glutathione content (d) in the kidney tissue were performed by kits. The expression of GPX-4 was performed by Western blot (e-f). ^*∗*^*P* < 0.05,^*∗∗*^*P* < 0.01, and ^*∗∗∗*^*P* < 0.001.

Folic acid induced AKI in mice, prompting a significant increase in the level of iron ions in the kidney tissue (*P* < 0.01). As illustrated in Figure 2(a), after 1 mg/kg ruscogenin treatment, iron ion levels gradually returned to normal levels, with substantial differences compared to the model group (*P* < 0.01). In addition, as depicted in Figures 2(b) and 2(c), malondialdehyde (MDA) and carbonylated protein levels in kidney tissues were significantly increased after being treated with folic acid (*P* < 0.001), indicating that ferroptosis occurred in kidney tissue cells. In contrast, MDA and carbonylated protein expression in the kidney were effectively controlled after ruscogenin treatment. Intracellular GSH content and GPX-4 expression were suppressed in kidney tissue cells after folic acid-induced iron death. This loss was reversed with ruscogenin treatment, which resulted in effective replenishment of GSH and GPX-4 (Figures 2(d)–2(f)). It is suggested that ruscogenin elements can inhibit folic acid-induced iron death in mouse kidney tissues.

### 3.3. Ruscogenin Inhibits Rev-Erb*α*/*β* Expression and Promotes Slc7a11 and HO-1 Expression

**Figure 3:**
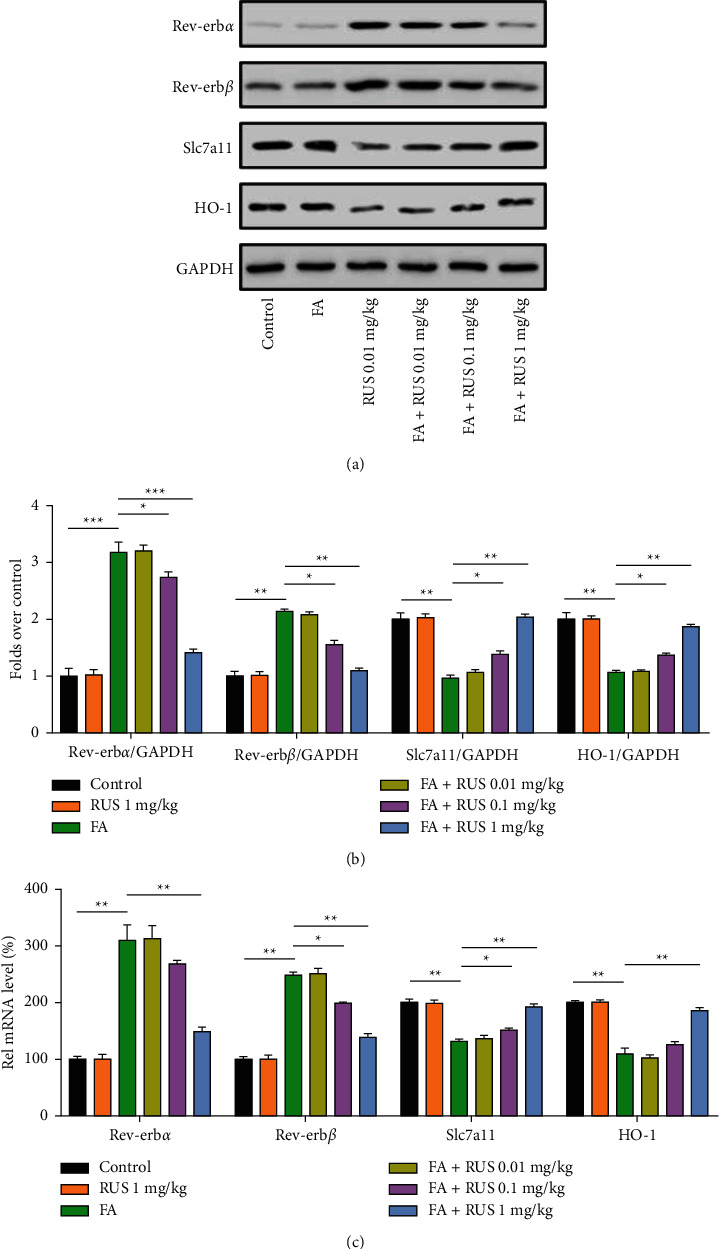
Ruscogenin inhibits Rev-erb*α*/*β* expression while increasing Slc7a11 and HO-1 expression. Western blotting was used to detect the expression of Rev-erb*α*/*β*, Slc7a11, and HO-1 protein in kidney tissues (a-b). q-PCR was used to detect Rev-erb*α*/*β*, Slc7a11, and HO-1 (c). ^*∗*^*P* < 0.05,^*∗∗*^*P* < 0.01,^*∗∗∗*^*P* < 0.001.

Rev-erb*α*/*β* is a nuclear transcriptional repressor that promotes ferroptosis by negatively regulating the expression of intracellular Slc7a11 and HO-1 proteins. The results are shown in Figure 3, and in folic acid-induced AKI tissues, the expression of Rev-erb*α*/*β* was increased in protein and mRNA level, while the level of Slc7a11 and HO-1 was suppressed. This suggests that folic acid may lead to repression of Slc7a11 and HO-1 transcription by inducing high expression of Rev-erb*α*/*β*. After treatment with ruscogenin, folic acid-induced expression of Rev-erb*α*/*β* was inhibited, leading to the recovery of their intracellular Rev-erb*α*/*β* content. At the same time, Slc7a11 and HO-1 protein expression was restored, suggesting that their repressed state was lifted. It was shown that ruscogenin could increase the levels of Slc7a11 and HO-1 by suppressing Rev-erb*α*/*β*, thereby inhibiting the occurrence of folic acid-induced iron death in kidney tissue cells and preventing the development of acute kidney injury under pathological conditions.

## 4. Discussion

**Figure 4:**
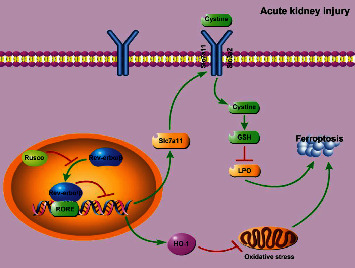
Ruscogenin's signaling pathway for inhibiting Rev-erb*α*/*β*-mediated cellular ferroptosis.

Rev-erb*α*/*β* is an important regulator of folic acid-induced AKI. By directly binding to RORE cis-elements, Rev-erb*α*/*β* may block the transcription of Slc7a11 and HO-1, two iron death repressor genes, increasing ferroptosis and AKI. Studies have shown that Rev-erb*α*/*β* deletion or inhibition significantly ameliorates folate-induced AKI [7]. In addition, suppression of Rev-erb*α*/*β* may improve other pathological conditions such as Alzheimer's disease [14], hyperhomocysteinemia [15], and hypercholesterolemia [16]. Therefore, the use of Rev-erb*α*/*β* as an intervention target for drug development and the elucidation of the mechanism of action of active ingredients in Chinese medicine is an extremely valuable strategy. In this study, Rev-erb*α*/*β* was used as the intervention target, and the inhibitory effect of ruscogenin on Rev-erb*α*/*β* was investigated. The results showed that in a folic acid-induced AKI model in mice, ruscogenin significantly reduced Rev-erb*α*/*β* expression, which in turn regulated the ferroptosis signaling pathway and exerted anti-AKI effects (Figure 4). Ferroptosis is one of the results of oxidative stress in the body, and it has been demonstrated that Ruscogenin can reduce oxidative stress and exert anti-inflammatory effects [17], and meanwhile, its antiferroptosis mechanism remains unknown. We demonstrated in this study that ruscogenin may exert antiferroptosis effects by downregulating the expression of Rev-erb*α*/*β* in pathological states, thus identifying a new anti-inflammatory mechanism of ruscogenin.

Using a folic acid-induced AKI model in mice, we studied the protective role of ruscogenin in modulating Rev-erb*α*/*β* in kidney damage. Folic acid-induced AKI has been clinically validated, and increased folic acid can lead to major AKI symptoms such as renal cell regeneration, renal cell death, and inflammation [18]. Because ferroptosis is the most common cause of folic acid-induced AKI, we chose the pathological phenomena of renal cell ferroptosis to examine ruscogenin's pharmacological mechanism based on its anti-AKI characteristics. Rev-erb*α*/*β*, an intervention target related to ferroptosis, was investigated, and its downstream signaling was examined when ruscogenin inhibited the onset of ferroptosis. However, it is still unknown about how ruscogenin elements regulate the expression of Rev-erb*α*/*β*. In addition to folic acid-induced AKI, ferroptosis is involved in the development of cisplatin, rhabdomyolysis, and ischemia/reperfusion-induced AKI [19–21]. This suggests that the therapeutic strategy of ruscogenin to alleviate folic acid-induced AKI *via* Rev-erb*α*/*β* protein may also apply to the other kinds of AKI discussed above. Folate-induced AKI is linked to the production of folate crystals in the renal tubules, according to research [22]. Although folic acid has direct nephrotoxic effects, it is unknown if ruscogenin influences the production of folic acid crystals.

Slc7a11 is one of the subunits of systemic xc- that mediates cystine uptake, and its mechanism of roles in the regulation of ferroptosis has been widely agreed upon. Regulation of Slc7a11 expression can be considered as the exact mechanism of ferroptosis inhibition by ruscogenin. Yet, the function of HO-1 in ferroptosis is currently controversial. A study by Kwon et al. showed that HO-1 expressed in lung fibroblasts greatly exacerbated erastin-induced ferroptosis [23]. However, Adedoyin et al. showed that HO-1 attenuated ferroptosis in renal proximal tubular cells and that HO-1 was protective against renal injury [24]. A study by Bolisetty et al. confirmed that induction of HO-1 expression in hepatoma cells treated with the ferroptosis inducer erastin had an inhibitory effect on the formation of ferroptosis [25]. The present study showed that the amelioration of iron death by ruscogenin was accompanied by elevating the level of HO-1, suggesting that increased HO-1 expression by ruscogenin may be one of the mechanisms by which it inhibits ferroptosis to exert its protective effect.

In conclusion, ruscogenin (1 mg/kg) significantly ameliorated folic acid-induced AKI in mice. The mechanism of ruscogenin to inhibit AKI is by decreasing the expression of Rev-erb*α*/*β*, increasing intracellular HO-1 and Slc7a11 levels, and ultimately inhibiting cellular iron death. Our study not only contributes to the understanding of the anti-inflammatory and anti-injury pharmacodynamic mechanisms of ruscogenin but also provides a new strategy for the treatment of AKI, which has some potential for clinical application.

## Figures and Tables

**Table 1:** The primer sequences.

	Forward (5′-3′)	Reverse (5′-3′)
Rev-erb*α*	TTTTTCGCCGGAGCATCCAA	ATCTCGGCAAGCATCCGTTG
Rev-erb*β*	GGAGTTCATGCTTGTGAAGGCTGT	CAGACACTTCTTAAAGCGGCACTG
Slc7a11	GGCACCGTCATCGGATCAG	CTCCACAGGCAGACCAGAAAA
HO-1	CACAGCACTATGTAAAGCGTCT	GTAGCGGGTATATGCGTGGG
Kim-1	GTTAAACCAGAGATTCCCACACG	TCTCATGGGGACAAAATGTAGTG

## Data Availability

The datasets used and analyzed during the current study are available from the corresponding author on reasonable request.
